# Giant Optical Anisotropy in a Covalent Molybdenum Tellurite via Oxyanion Polymerization

**DOI:** 10.1002/advs.202306670

**Published:** 2024-01-30

**Authors:** Tianhui Wu, Xingxing Jiang, Kaining Duanmu, Chao Wu, Zheshuai Lin, Zhipeng Huang, Mark G. Humphrey, Chi Zhang

**Affiliations:** ^1^ China‐Australia Joint Research Center for Functional Molecular Materials School of Materials Science and Engineering Ocean University of China Qingdao 266404 China; ^2^ State Key Laboratory of Metastable Materials Science and Technology Hebei Key Laboratory of Applied Chemistry Yanshan University Qinhuangdao 066004 China; ^3^ School of Chemical Science and Engineering Tongji University Shanghai 200092 China; ^4^ Technical Institute of Physics and Chemistry Chinese Academy of Sciences Beijing 100190 China; ^5^ Research School of Chemistry Australian National University Canberra ACT 2601 Australia

**Keywords:** birefringent crystal, molybdenum tellurite, optical anisotropy, second‐order Jahn–Teller effect, structure‐property relationships

## Abstract

Large birefringence is a crucial but hard‐to‐achieve optical parameter that is a necessity for birefringent crystals in practical applications involving modulation of the polarization of light in modern opto‐electronic areas. Herein, an oxyanion polymerization strategy that involves the combination of two different types of second‐order Jahn–Teller distorted units is employed to realize giant anisotropy in a covalent molybdenum tellurite. Mo(H_2_O)Te_2_O_7_ (MTO) exhibits a record birefringence value for an inorganic UV‐transparent oxide crystalline material of 0.528 @ 546 nm, which is also significantly larger than those of all commercial birefringent crystals. MTO has a UV absorption edge of 366 nm and displays a strong powder second‐harmonic generation response of 5.4 times that of KH_2_PO_4_. The dominant roles of the condensed polytellurite oxyanions [Te_8_O_20_]^8−^ in combination with the [MoO_6_]^6−^ polyhedra in achieving the giant birefringence in MTO are clarified by structural analysis and first‐principles calculations. The results suggest that polymerization of polarizability‐anisotropic oxyanions may unlock the promise of birefringent crystals with exceptional birefringence.

## Graphical abstract

A covalent molybdenum tellurite, Mo(H_2_O)Te_2_O_7_, which exhibits significantly large birefringence of exp. 0.528 @ 546 nm—being the largest value for inorganic UV‐transparent oxides, is successfully constructed by an oxyanion polymerization strategy.

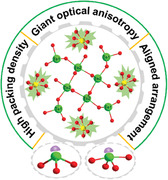

## 1 Introduction

Birefringent materials capable of modulating polarized light are of great importance in many fields of modern opto‐electronic technology such as linear optical devices, fiber optic sensors, and advanced optical communication systems.^[^ ^1^, ^2^ ^]^ Several well‐known inorganic birefringent materials have been discovered, such as natural crystals (e.g., CaCO_3_ (Δ*n* = 0.175 @ 533 nm)^[^ ^3a^ ^]^ and TiO_2_ (Δ*n* = 0.299 @ 577 nm))^[^ ^3b^ ^]^ and artificial crystals (e.g., *α*‐BaB_2_O_4_ (Δ*n* = 0.142 @ 533 nm),^[^ ^3c^ ^]^ YVO_4_ (Δ*n* = 0.209 @ 1064 nm),^[^ ^3d^ ^]^ and LiNbO_3_ (Δ*n* = 0.083 @ 632.8 nm)).^[^ ^3e^ ^]^ Unfortunately, drawbacks, such as inadequate crystal quality due to defects and impurities in natural crystals, significant anisotropic thermal expansion in *α*‐BaB_2_O_4_, weak UV transmittance in YVO_4_, and the relatively low birefringence of LiNbO_3_,^[^ ^3^ ^]^ have significantly restricted their applications in high‐tech demands. New birefringent materials with strong optical anisotropy are consequently urgently required.

Birefringence (or optical anisotropy) results when light travels with different speeds along crystallographic directions with distinctly different microstructures.^[^ ^4^ ^]^ A structural unit with large polarizability anisotropy is consequently a prerequisite for materials to obtain a large birefringence.^[^ ^5^ ^]^ Non‐π‐conjugated oxyanions (e.g., [SO_4_]^2−^, [PO_4_]^3−^, and [SiO_4_]^4−^)^[^ ^6^ ^]^ possess intrinsically small polarizability anisotropy and are therefore unsuitable. One effective strategy to afford a strong optical anisotropy involves the incorporation of inorganic π‐conjugated planar oxyanions (e.g., [BO_3_]^3−^, [CO_3_]^2−^, and [NO_3_]^−^)^[^ ^7^ ^]^ into molecular structures, examples including *α*‐BaB_2_O_4_,^[^ ^3e^ ^]^ CaCO_3_,^[^ ^3a^ ^]^ and Ca(BO_2_)_2_.^[^ ^2b^ ^]^ The birefringence induced by these structural units is usually below 0.2 @ 1064 nm. In contrast to these π‐conjugated oxyanions, *d* ^0^ transition metal (TM) cations (e.g., Mo^6+^, W^6+^, V^5+^, and Nb^5+^)^[^ ^8^ ^]^ and lone‐pair electron cations (e.g., Sn^2+^, Pb^2+^, Te^4+^, and I^5+^)^[^ ^9^ ^]^ may increase the birefringence because of their larger polarizability anisotropy induced by second‐order Jahn–Teller (SOJT) distortion. Although many optical crystals with enhanced birefringence,^[^ ^10^ ^]^ such as *α*‐BaTeMo_2_O_9_,^[^ ^10a^ ^]^ Rb_2_VO(O_2_)_2_F,^[^ ^10b^ ^]^ RbTeMo_2_O_8_F,^[^ ^10c^ ^]^ K_2_SbC_2_O_4_Cl_3_,^[^ ^10d^ ^]^ and RbSn_2_Cl_5_,^[^ ^10e^ ^]^ have been reported, these extant materials always exhibit wide variability in birefringence with typical values lower than 0.3. An effective structural design strategy has yet to be developed for the creation of high‐performance birefringent crystals.

The key to achieve large birefringence lies with high packing density and the aligned arrangement of the structural units that possess large polarizability anisotropy in a crystal lattice.^[^ ^11^ ^]^ Of the possible crystalline constitutions, large optical anisotropy can be achieved in inorganic oxides^[^ ^12a^ ^]^ in which appropriate oxyanions as well as their connection modes are carefully selected. In the present study, we propose an oxyanion polymerization strategy, in which three types of polarizability‐anisotropic structural units^[^ ^8^, ^9^ ^]^ are employed in the construction of a condensed structure to maximize the optical anisotropy and achieve a large birefringence. In stark contrast to alkali/alkali‐earth counter‐cations,^[^ ^12b^ ^]^ SOJT polyhedra exhibiting maximized local polarizability anisotropy are usually regarded as the preferred structural units in the design of optical materials with sufficient birefringence.^[^ ^4b^ ^]^ Materials constructed solely from SOJT‐active cations without introducing alkali/alkali‐earth counter‐cations are beneficial in the pursuit of an optically anisotropic structure, owing to the resultant high‐density of the SOJT‐active cations. Unlike isolated SOJT oxyanions, highly polymerized SOJT oxyanions can facilitate a relatively condensed structure, and this may afford a large local anisotropic polarizability.^[^ ^12c^ ^]^ By following the strategy outlined above, we have found that a combination of polymerized tellurium anions and molybdenum polyhedra leads to a new covalent molybdenum tellurite Mo(H_2_O)Te_2_O_7_ (MTO) that exhibits the largest birefringence for an inorganic UV‐transparent oxide crystal (Δ*n* = 0.528 @ 546 nm), even surpassing those of the commercial birefringent crystals YVO_4_ and TiO_2_. Furthermore, a UV absorption edge of 366 nm and a strong powder second‐harmonic generation (SHG) response of 5.4 × KH_2_PO_4_ (KDP) are observed for noncentrosymmetric MTO. The role of the polymerized functional units in enhancing the linear and nonlinear optical responses has been clarified by crystal structure analysis and first‐principles simulations. This study not only affords a new birefringent crystal that is potentially of use in practical optical device applications but also unveils a new perspective for the development of high‐performance next‐generation birefringent crystalline materials.

## 2 Results and Discussion

The title compound MTO was synthesized by a straightforward hydrothermal method, with a mixture of MoO_3_, TeO_2_, CeO_2_, HF, and deionized water at 230 °C. The structure was determined by single‐crystal X‐ray diffraction (details are provided in the Supporting Information) and the purity of MTO was confirmed by powder X‐ray diffraction studies (Figure S1, Supporting Information). Its chemical composition was determined by energy‐dispersive X‐ray diffraction (Figure S2, Supporting Information), with the results matching well with the formula suggested by the single‐crystal X‐ray diffraction data. Thermogravimetric analysis of MTO over the temperature range of 30–800 °C under N_2_ revealed that MTO is thermally stable at 240 °C (Figure S3, Supporting Information). When MTO was further heated, the weight loss over the temperature range 240–340 °C was 3.74%, corresponding to the removal of one H_2_O molecule (calculated value: 3.74%). No further weight loss was observed over the temperature range 340–800 °C.

UV–Vis‐NIR transmittance measurement of block single crystals of MTO reveals an absorption edge of 366 nm (Figure S4, Supporting Information), corresponding to a bandgap of 3.39 eV. This value is slightly larger than those of other tellurite‐based *d* ^0^‐TM optical crystals^[^ ^13^ ^]^ such as Ag_2_Te_3_Mo_3_O_16_ (2.85 eV),^[^ ^13a^ ^]^ *β*‐BaTeMo_2_O_9_ (2.95 eV),^[^ ^13b^ ^]^ Na_2_Te_3_Mo_3_O_16_ (2.95 eV),^[^ ^13c^ ^]^ and *α*‐BaTeMo_2_O_9_ (3.12 eV).^[^ ^10a^ ^]^ The Infrared (IR) spectrum of MTO was measured over the rangel 4000–400 cm^−1^ at room temperature, the assignments of the IR absorption peaks being listed in Figure S5, Supporting Information. The absorption bands observed between 935 and 888 cm^−1^ are assigned to the stretching vibrations of the [MoO_6_]^6−^ units, those from 820 to 690 cm^−1^ are attributed to Te─O stretching vibrations, and the peaks between 600 and 400 cm^−1^ are derived from Te─O─Te vibrations. Characteristic absorption bands at 3363 and 1621 cm^−1^ confirm the presence of H_2_O molecules in MTO.

**Figure 1:**
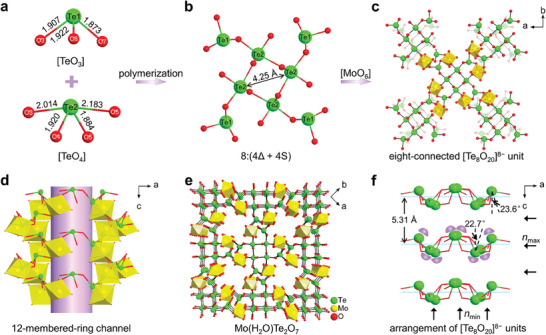
a) [TeO_3_]^2−^ and [TeO_4_]^4−^ units. b) [Te_8_O_20_]^8−^ polytellurite anion in Mo(H_2_O)Te_2_O_7_. c) Linking mode of the [Te_8_O_20_]^8−^ unit of Mo(H_2_O)Te_2_O_7_ and eight adjacent units. d) View of the 12‐membered‐ring channel in Mo(H_2_O)Te_2_O_7_. e) Perspective view of the 3D covalent framework of Mo(H_2_O)Te_2_O_7_ along the *c*‐axis. Hydrogen atoms and hydrogen bonds are omitted for clarity. f) Arrangement of the [Te_8_O_20_]^8−^ polytellurite anion in Mo(H_2_O)Te_2_O_7_. Purple crescents indicate the approximate position of lone‐pair electrons. Up and left arrows indicate the direction of the minimum and maximum refractive indices along the *c*‐axis and *a*‐axis, respectively.

MTO crystallizes in the tetragonal system within the noncentrosymmetric space group *I*‐4 (no. 82: Tables S1–S4, Supporting Information). In the molybdenum tellurite crystal, the primary building unit, the polytellurite oxyanion [Te_8_O_20_]^8−^, is composed of four [Te(1)O_3_] units (∆) and four [Te(2)O_4_] seesaws (S) linked via corner‐sharing oxygens [O(3) and O(5)] (**Figure** **1**a). The unique polytellurite oxyanion can be written as [8:(4∆ + 4S)] (Figure 1b), the first time that this has been observed in the tellurite system.^[^ ^14^ ^]^ The distance between adjacent polytellurite anion planes is 5.31 Å (Figure 1f). Each [Te_8_O_20_]^8−^ is attached to eight adjacent [Te_8_O_20_]^8−^ via [MoO_6_]^6−^ octahedral linkages (Figure 1c). Further connectivity of the polytellurite anion [Te_8_O_20_]^8−^, namely sharing [MoO_6_]^6−^ octahedra, yields a 3D covalent framework in the *ab* plane with 12‐membered‐ring channels (Figure 1d,e).

In each asymmetric unit of MTO, there are two independent Te atoms, one Mo atom, eight O atoms, and two H atoms (Figure S6a, Supporting Information). Te(1) adopts a trigonal pyramidal geometry, while Te(2) exhibits a seesaw geometry (Figure 1a). The Te─O bond distances of the [TeO_3_]^2−^ and [TeO_4_]^4−^ units are in the ranges 1.873(6)–1.922(6) Å and 1.884(6)–2.183(6) Å, respectively. The Mo^6+^ cation adopts a 6‐coordinate environment ([MoO_6_]^6−^), with a wide range of Mo─O bond lengths [1.662(6) to 2.349(8) Å] (Figure S6b, Supporting Information). The [MoO_6_]^6−^ octahedron displays a strong out‐of‐center distortion toward an edge along the *C*2 direction (Δ*d* = 1.12),^[^ ^15^ ^]^ and this is immensely beneficial for achieving large local microscopic polarizability and consequently enhancement of the SHG response. Both Te^4+^ and Mo^6+^ cations are in asymmetric coordination environments, attributable to SOJT effects. Most importantly, the [TeO_3_]^2−^ and [TeO_4_]^4−^ units in the polytellurite oxyanion [Te_8_O_20_]^8−^ maintain an almost coplanar arrangement by linking with [MoO_6_]^6−^ polyhedra (Figure 1c), and the lone‐pair electrons of the Te^4+^ cation in the [TeO_3_]^2−^ and [TeO_4_]^4−^ units consequently align in approximately the same direction along the *a*‐axis (Figure 1f), a favorable mode for generating strong optical anisotropy.

**Figure 2:**
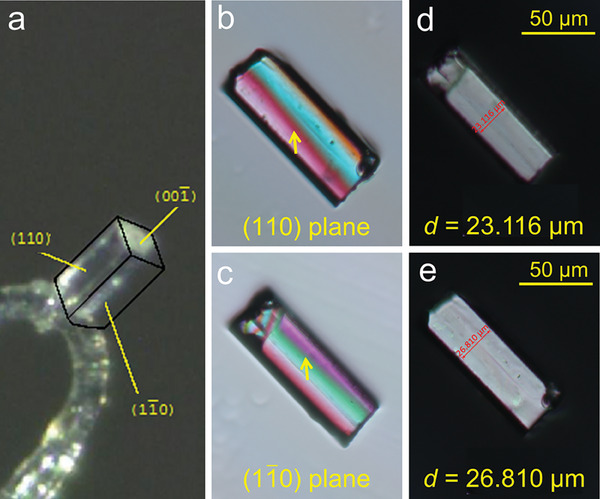
a) Crystal orientation of a Mo(H_2_O)Te_2_O_7_ crystal indexed by single‐crystal X‐ray diffraction. A (110) crystal plate (b) and ($1\bar{1}0$) crystal plate (c) of Mo(H_2_O)Te_2_O_7_ achieving extinction. The corresponding thickness of a (110) crystal plate (d) and ($1\bar{1}0$) crystal plate (e) for birefringence measurements.

**Figure 3:**
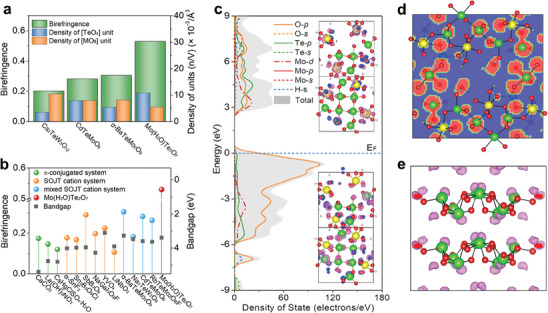
a) Birefringence and density of the functional units for Mo(H_2_O)Te_2_O_7_, Cs_2_TeW_3_O_12_, CdTeMoO_6_, and *α*‐BaTeMo_2_O_9_. b) Birefringence and bandgap for Mo(H_2_O)Te_2_O_7_, and some representative UV‐transparent optical materials with bandgaps in the range of 3.1 to 6.2 eV. c) Total density of states and partial density of states of Mo(H_2_O)Te_2_O_7_; Fermi levels (dotted lines) located at zero. The insets are electron density maps of the VBM (lower) and CBM (upper) in the primitive cell for Mo(H_2_O)Te_2_O_7_. The isosurface value is set to 0.001 eV Å^−3^. Red: oxygen; yellow: molybdenum; green: tellurium; gray: hydrogen. d) Sliced‐plane of the ELF diagram for Mo(H_2_O)Te_2_O_7_ along the [001] direction. e) ELF diagram associated with the polytellurite anion [Te_8_O_20_]^8−^ along the [100] direction, in which asymmetric lobe‐like isosurface (pink) near each Te^4+^ center is observed.

To quantify its strong optical anisotropy, a birefringence measurement was performed using a high‐quality cuboid single crystal under a cross‐polarizing microscope equipped with a Berek compensator at 546 nm.^[^ ^16^ ^]^ Single‐crystal X‐ray diffraction was employed to determine the crystal planes for MTO; (110), ($1\bar{1}0$), and ($00\bar{1}$) are clearly observed (**Figure** **2**a). For the birefringence measurement on the (110) crystal plane, the optical path difference at 546 nm was determined to be 12.20 µm with a thickness of 23.116 µm (Figure 2b,d). The derived birefringence of MTO for the (110) crystal plane is 0.528 @ 546 nm according to the formula *R* = *d* × Δ*n*, where *R* represents the optical path difference and *d* denotes the thickness. The same birefringence measurement on the ($1\bar{1}0$) crystal plane was also performed (Figure 2c,e). Given the optical path difference of 14.12 µm and thickness of 26.810 µm, the birefringence of MTO on the ($1\bar{1}0$) plane is 0.527 @ 546 nm, coinciding with the birefringence characteristics of a uniaxial crystal. The refractive indexes of MTO along different crystal axes were calculated using the CASTEP package (Figure S7, Supporting Information). MTO exhibits unusually strong optical anisotropy with a calculated birefringence of 0.35 @ 546 nm, smaller than the experimental value. The error between theoretical and experimental birefringence values is similar to previous reports.^[^ ^5^, ^6^, ^7^, ^9^ ^]^ The birefringence of MTO is significantly larger than those of the commercially available TM‐based birefringent crystals YVO_4_ (0.209 @ 1064 nm)^[^ ^3d^ ^]^ and LiNbO_3_ (0.083 @ 632.8 nm).^[^ ^3e^ ^]^ In addition, its experimental value not only exceeds the birefringences of all reported TM tellurites (*α*‐BaTeMo_2_O_9_ (0.305 @ 404.7 nm),^[^ ^10a^ ^]^ Na_2_W_2_TeO_9_ (0.1823 @ 532 nm),^[^ ^17a^ ^]^ CdTeMoO_6_ (0.2818 @ 514 nm),^[^ ^17b^ ^]^ and RbTeMo_2_O_8_F (0.263 @ 546 nm)),^[^ ^10c^ ^]^ but is also superior to those of the recently reported inorganic UV‐transparent optical crystals La(OH)_2_NO_3_ (0.146 @ 589.6 nm),^[^ ^18^ ^]^ CsHgClSO_4_·H_2_O (0.12 @ 546 nm),^[^ ^5b^ ^]^ Sn_2_B_5_O_9_Cl (0.168 @ 546 nm),^[^ ^9d^ ^]^ and SbB_3_O_6_ (0.290 @ 546 nm)^[^ ^9e^ ^]^ (**Figure** **3**b).^[^ ^4b^ ^]^

The unusually large birefringence of MTO can be definitively attributed to its 3D covalent framework, which consists of three types of polarizability‐anisotropic structural units ([TeO*_x_*] (*x* = 3, 4) and [MoO_6_]^6−^) without conventional alkali/alkali‐earth counter‐ions. To investigate the optical anisotropic property contributions from the different structural units in MTO, a real‐space atom cutting technique was utilized, the calculated contributions being listed in Table S5, Supporting Information. The contributions are mainly from the [TeO_3_]^2−^ (40.5%) and [TeO_4_]^4−^ (37.7%) units, rather than the [MoO_6_]^6−^ (17.4%) units and the H_2_O molecules. The largest contribution comes from the [TeO_3_]^2−^ and [TeO_4_]^4−^ groups, demonstrating that introducing highly polymerized SOJT oxyanions, that is, the polytellurite oxyanion [Te_8_O_20_]^8−^, to modulate the structure and function of oxides can be a promising way to develop birefringent materials. To clearly demonstrate the influences of the polymerized SOJT oxyanions on the optical anisotropy from a structural perspective, we first calculated the density of the lone‐pair electron cation Te^4+^ in extant optical *d* ^0^‐TM tellurites (Figure 3a and **Table** **1**). The density of the lone‐pair electron cation Te^4+^ in MTO (0.0108 Å^−3^) is the highest for an optical *d* ^0^‐TM tellurites, and this can be attributed to the presence of the polytellurite oxyanion [Te_8_O_20_]^8−^. Second, the arrangement of the [TeO_x_] units in MTO was quantified from bond valence calculations. We define *θ* as the angle between the direction of the [TeO*_x_*] maximum component's dipole moment and the direction of the total dipole moment. In principle, the smaller this angle is, the more uniform the alignment of the lone‐pair electron on [TeO*_x_*] units will be. The angles *θ* between the direction of the total dipole moment and the direction of the maximum component dipole moments for [TeO_3_]^2−^ and [TeO_4_]^4−^ are 23.6° and 22.7° (Figure 1f), respectively. These values are significantly smaller than those of *α*‐BaTeMo_2_O_9_^[^ ^10a^ ^]^ (30.2° and 29.9°) (Table S6, Supporting Information) which has the largest reported birefringence value (Δ*n* = 0.305 @ 404.7 nm) for an extant optical *d* ^0^‐TM tellurite. The [TeO*_x_*] units in MTO are almost uniformly arranged in the *ac* plane, and a larger optical anisotropy can be anticipated for MTO compared to those of other *d* ^0^‐TM tellurites (e.g., *α*‐BaTeMo_2_O_9_,^[^ ^10a^ ^]^ Cs_2_TeW_3_O_12_,^[^ ^19^ ^]^ and CdTeMoO_6_)^[^ ^17b^ ^]^ as a result of the high‐density of [TeO*_x_*] units, as well as their favorable aligned arrangement.

**Table 1:** Experimental birefringences and densities of lone‐pair cations (n V^−1^) (×10^−3^/Å^3^) for optical *d* ^0^‐TM tellurites.

Compound	Space group	Birefringence	*ρ* [n V^−1^]
PbVTeO_5_F^[^ ^17c^ ^]^	*Pbca*	0.142 @ 590 nm	7.66
LiNbTeO_5_^[^ ^17d^ ^]^	*P*2_1_	0.083 @ 632.8 nm	9.76
*α*‐BaTeMo_2_O_9_^[^ ^10a^ ^]^	*Pca*2_1_	0.305 @ 404.7 nm	5.37
*β*‐BaTeMo_2_O_9_^[^ ^13b^ ^]^	*P*2_1_	0.226 @ 435 nm	5.49
*α*‐BaW_2_TeO_9_^[^ ^17e^ ^]^	*Pnma*	0.247 @ 404.7 nm	5.20
Cs_2_TeMo_3_O_12_^[^ ^17f^ ^]^	*P*6_3_	0.184 @ 546.1 nm	3.46
Cs_2_TeW_3_O_12_^[^ ^19^ ^]^	*P*6_3_	0.201 @ 546.1 nm	3.47
RbTeMo_2_O_8_F^[^ ^10c^ ^]^	*Pn*	0.263 @ 546 nm	5.11
Zn_2_MoTeO_7_^[^ ^17g^ ^]^	*P*2_1_	0.084 @ 532 nm	8.45
CdTeMoO_6_^[^ ^17b^ ^]^	*P*2_1_ *m*	0.287 @ 514 nm	7.90
Cd_3_WTe_2_O_10_^[^ ^17h^ ^]^	*P*2_1_	0.11 @ 550 nm	8.24
Na_2_W_2_TeO_9_^[^ ^17a^ ^]^	*Cc*	0.182 @ 532 nm	5.31
Na_6_W_6_Te_4_O_29_^[^ ^17a^ ^]^	*P*2_1_/*c*	0.109 @ 532 nm	6.68
Mo(H_2_O)Te_2_O_7_^this work^	*I*	0.528 @ 546 nm	10.80

In order to develop an in‐depth understanding of the exceptional birefringence of MTO, theoretical calculations were performed using the CASTEP package. Electronic bandgap predictions by density functional theory (DFT) gave an indirect bandgap of 2.92 eV for MTO (Figure S8, Supporting Information), which is smaller than the experimental result, an outcome attributed to the derivative discontinuity of exchange‐correlation energy. The contributions of various atoms and their orbitals to other bands around the Fermi level can be seen from the density of states plots in Figure 3c. The top of the valence band (VB) is dominated by O‐2p bonding states, as well as Te‐5p and Mo‐4d orbitals, and in the bottom of the conduction zone, the dominant orbital contributions come from Te‐5p, Mo‐4d, and O‐2p orbitals. The electronic states of the Te and Mo atoms overlap well with those of the O atoms from the valence band to the conduction band (CB), implying strong covalent interactions in the Te─O and Mo─O bonds. These results indicate that a combination of interactions among the [TeO_3_]^2−^, [TeO_4_]^4−^, and [MoO_6_]^6−^ units are responsible for the linear optical (i.e., birefringence) and nonlinear optical (i.e., SHG) properties of MTO. To visualize such contributions, the valence band maximal (VBM) and conduction band minimal (CBM) densities of states in the primitive cell for MTO are shown in the insets of Figure 3c. The VBM is composed of O‐2p and Te‐5p states localized in the [TeO_3_]^2−^ and [TeO_4_]^4−^ units, while the CBM is largely composed of states from the [MoO_6_]^6−^ units.

We also analyzed electron localization function (ELF) maps of MTO. The (001)‐projected ELF map shows the synergy between the [TeO_3_]^2−^, [TeO_4_]^4−^, and [MoO_6_]^6−^ units (Figure 3d). In stark contrast to the electron density around the Mo atoms, the electron localization around the Te atoms is highly asymmetric owing to its lone‐pair electron, and so they make a significant contribution to the optical properties. The directions of the lone‐pair electrons of the [TeO_3_]^2−^ and [TeO_4_]^4−^ units oppose and are almost parallel, viewed along the [100] direction (Figure 3e), thereby making a strongly positive contribution to the birefringence of MTO.

Motivated by the noncentrosymmetric structure of MTO, the powder SHG response was assayed by the Kurtz and Perry method. MTO exhibits an SHG response of about 5.4 times that of KDP at 1064 nm laser radiation (particle size range 105–150 µm) (Figure S9a, Supporting Information). The SHG intensity signal gradually increases with particle size and finally reaches a constant value (Figure S9b, Supporting Information), indicating the phase‐matching behavior of MTO at 1064 nm. The calculated shortest SHG phase‐matching wavelength of MTO is 435 nm (Figure S7, Supporting Information) The powder SHG response of MTO is significantly larger than those of reported tellurite‐based crystals, such as *α*‐BaTeMo_2_O_9_ (0.2 × KDP),^[^ ^10a^ ^]^ *β*‐BaTeMo_2_O_9_ (2.5 × KDP),^[^ ^20a^ ^]^ BaF_2_TeF_2_(OH)_2_ (3 × KDP),^[^ ^20b^ ^]^ TlTeVO_5_ (40 × *α*‐SiO_2_),^[^ ^20c^ ^]^ and Li_2_ZrTeO_6_ (2.5 × KDP).^[^ ^20d^ ^]^ The SHG coefficients *d*_ij_ were calculated by the method of Lin et al.^[^ ^21^ ^]^ The nonzero independent SHG coefficients (*d*_14_ and *d*_15_) under the restriction of Kleinman symmetry^[^ ^22a^ ^]^ are as follows: *d*_14_ = 4.28 pm V^−1^ and *d*_15_ = − 2.34 pm V^−1^ (Table S5, Supporting Information). The calculated average SHG coefficient is slightly larger than the experimental result, which can be attributed to the fact that the SHG calculations are based on an optimized single‐crystal structure that may generate a larger SHG effect. From a structural perspective, the large SHG response of MTO is closely related to the cooperative effect of the [MoO_6_]^6−^, [TeO_3_]^2−^, and [TeO_4_]^4−^ units on the basis of anionic group theory.^[^ ^22b^ ^]^ Specifically, the [MoO_6_]^6−^ octahedron in MTO displays a strong out‐of‐center distortion toward an edge along the *C*2 direction, and these [MoO_6_]^6−^ octahedra stack uniformly within a unit cell (Figure S6c, Supporting Information). The spatial arrangement of the [MoO_6_]^6−^ octahedra with large local microscopic polarizability is beneficial for the enhancement of the SHG response. Although the lone‐pair electrons on the [TeO_3_]^2−^ and [TeO_4_]^4−^ units are arranged in almost opposing directions (Figure 1f), the superimposed microscopic polarizability of the [TeO_3_]^2−^ and [TeO_4_]^4−^ units (with significant microscopic polarizabilities) also contribute to the global effect. The microscopic second‐order susceptibilities associated with the [MoO_6_]^6−^, [TeO_3_]^2−^, and [TeO_4_]^4−^ units constructively reinforce, leading to a large optical nonlinearity, which is confirmed by the powder SHG measurements on the crystalline MTO. The origin of the SHG response was explored further via an SHG‐weighted electron density analysis. The SHG‐weighted electron clouds of *d*_14_ are mainly located on the [TeO_3_]^2−^, [TeO_4_]^4−^, and [MoO_6_]^6−^ units (Figure S10a,b, Supporting Information). The Mo‐4*d* orbitals and to a lesser extent Te‐5*p* orbitals make the most significant contributions to the SHG response in the unoccupied states, while in the occupied states, the nonbonding O‐2p orbitals afford the primary contribution to the SHG response. The SHG contributions from all units were calculated using a real‐space atom‐cutting method, the [TeO_3_]^2−^, [TeO_4_]^4−^, and [MoO_6_]^6−^ units making the dominant contributions to the largest coefficient *d*_14_ and these units accounting for 39%, 28.7%, and 30.1% for MTO, respectively. These results indicate that the [TeO_3_]^2−^, [TeO_4_]^4−^, and [MoO_6_]^6−^ units are the main contributors to the SHG response of MTO under the perturbation of ambient optoelectronic fields.

## 3 Conclusion

In summary, the new molybdate tellurite MTO was synthesized by an oxyanion polymerization strategy and found to exhibit a strong SHG response (≈5.4 × KDP) and a UV absorption edge (366 nm). Its excellent NLO properties are attributed to cooperative contributions from the [TeO*_x_*] (*x* = 3, 4) and [MoO_6_]^6−^ units. Polymerization of the SOJT polyhedra [Te_8_O_20_] and [MoO_6_]^6−^ results in a giant birefringence (0.528 @ 546 nm), which is the largest for an inorganic UV‐transparent oxide crystal and exceeds those of commercial birefringent crystals. Theoretical calculations suggest that the remarkable linear optical properties of MTO are driven by a favorable ordered alignment of the high‐density [TeO*_x_*], as well as synergetic [MoO_6_]^6−^ polyhedra. The study demonstrates that giant optical anisotropy can be achieved by a combination of polymerized tellurium anions and molybdenum polyhedra and, accordingly, provides an outstanding paradigm for the future development of high‐performance birefringent crystalline materials.

## 4 Experimental Section

### Synthesis

Molybdenum trioxide (MoO_3_, 99.5%, Xiya Reagent), tellurium dioxide (TeO_2_, 99.8%, Xiya Reagent), cerium dioxide (CeO_2_, 99.99%, Xiya Reagent), and hydrofluoric acid (HF, 40%, Tansoole Chemical Reagent) were commercially available and used as received without further purification (Caution: hydrofluoric acid is toxic and corrosive! It must be handled with extreme caution and appropriate protective equipment and training). A mixture of TeO_2_ (0.319 g, 2 mmol), MoO_3_ (0.144 g, 1 mmol), CeO_2_ (0.103 g, 0.6 mmol), HF (0.2 mL), and deionized water (1 mL) was placed in a tightly sealed 23 mL autoclave equipped with a Teflon liner. The autoclave was heated at 230 °C for 72 h and then cooled slowly to room temperature at a rate of 4 °C h^−1^. The product was collected by vacuum filtration, washed with deionized water, and then dried in the air. Block colorless crystals of MTO were isolated in a yield of 65% (based on Te) using a microscope. The reaction starting materials and stoichiometry are critical for the synthesis of MTO. When the amount of starting material CeO_2_ is less than 0.5 mmol, MTO cannot be obtained. It is noteworthy that using a small amount of CeO_2_ and HF is conducive to enhancing the yield of the target compound. The pH of the solution also plays a vital role in the synthesis of compound MTO, with acidic conditions favoring its formation.

### Single‐Crystal Structure Determination

A block crystal of MTO with dimensions of 0.14 × 0.08 × 0.07 mm^3^ was selected for the single‐crystal structure determination. The diffraction data collection was carried out on a Bruker D8 VENTURE CMOS X‐ray source with Mo K*α* radiation (*λ* = 0.71073 Å) at 293(2) K. Data collection and reduction were performed using the APEX II software, and absorption corrections were implemented based on a multiscan‐type model. The crystal structure was solved by direct methods and refined on *F^2^* by full‐matrix least squares methods using the SHELXTL‐*97* software package.^[^ ^23^ ^]^ All non‐hydrogen atoms were refined with anisotropic displacement parameters. On the basis of the requirements of charge balance and bond valence calculations, O(1) was assigned as being in a water molecule. The absolute structure was examined for missing symmetry elements using PLATON, and none were found.^[^ ^24^ ^]^ Crystal data and structure refinement information for MTO are summarized in Table S1, Supporting Information. Atomic coordinates and equivalent isotropic displacement parameters, as well as calculated bond valence sums (BVS), are given in Table S2, Supporting Information, while selected bond distances and angles are collected in Table S3, Supporting Information. BVS calculations of 3.93–3.97, 6.39, and 1.73–2.30 for Te, Mo, and O atoms, respectively, are consistent with the expected valences. Hydrogen‐bonding interactions for MTO are listed in Table S4, Supporting Information.

### Powder X‐ray Diffraction

Powder XRD data for MTO were collected using a Bruker D8 Advance X‐ray diffractometer equipped with Cu K*α* radiation (*λ* = 1.5418 Å) with a 2*θ* range of 5°–70° and a scan step width of 0.02°.

### Energy‐Dispersive X‐ray Spectroscopy

Microprobe elemental analyses were performed using energy‐dispersive X‐ray spectroscopy (EDS) with a field‐emission scanning electron microscope (Hitachi S‐4800, Japan).

### UV–vis Transmission Spectrum

The UV–vis transmittance spectrum was measured at room temperature with crystal samples. Data were collected on a PerkinElmer Lambda‐950 UV/Vis/NIR spectrophotometer scanning in the range of 200–2500 nm.

### Infrared Spectrum

The IR spectrum was recorded on a Nicolet iS10 Fourier transform IR spectrometer (resolution 4 cm^−1^, spectral range 400–4000 cm^−1^).

### Thermal Stability

A Netzsch STA 409 PC thermal analyzer was used to analyze the thermal stability of MTO. The sample was heated from 30 to 800 °C with a heating rate of 15 °C min^−1^ in nitrogen gas.

### Birefringence Measurements

The birefringence of crystalline sample MTO was assessed with a polarizing microscope (ZEISS AXIO Scope.A1) equipped with a Berek compensator. The wavelength of the light source was 546 nm. The birefringence was calculated according to Equation (1)

(1) $$\Delta R\;\left(\mathrm{retardation}\right)\;=\;|n_{e}\;-\;n_{o}|\;\times \;T\;=\;\Delta n\;\times \;T$$

where Δ*R* denotes the optical path difference, Δ*n* represents the birefringence, and *T* is the thickness of the crystal. The positive and negative rotation of compensation affords the relative retardation. To improve the accuracy of the birefringence measurement, a transparent cuboid MTO crystal was chosen for the study.

### Second‐Harmonic Generation

Measurements of the powder frequency‐doubling effect were carried out by the method of Kurtz and Perry,^[^ ^25^ ^]^ employing a Q‐switched Nd:YAG laser at 1064 nm laser radiation. The crystal samples were ground and sieved into seven distinct particle size ranges (<26, 26–50, 50–74, 74–105, 105–150, 150–200, 200–280 µm), which were pressed into disks with a diameter of 6 mm that were placed between glass microscope slides and secured with tape in a 1 mm thick aluminum holder. Crystalline KH_2_PO_4_ (KDP) was ground and sieved into the same particle size ranges (<26, 26–50, 50–74, 74–105, 105–150, 150–200, 200–280 µm) which were used as references. The intensities of the frequency‐doubled output emitted from the crystalline samples were measured using a photomultiplier tube.

### Theoretical Calculations

First‐principles calculations on MTO were performed using the CASTEP package,^[^ ^26a^ ^]^ a total energy package based on pseudopotential DFT.^[^ ^26b,c^ ^]^ The correlation‐exchange terms in the Hamiltonian were described by the functional developed by PBE^[^ ^26d^ ^]^ in the generalized gradient approximation (GGA)^[^ ^26e^ ^]^ form. The optimized norm‐conserving pseudopotentials^[^ ^26f^ ^]^ were adopted to model the effective interaction between the valence electrons and atom cores, which allowed the choice of a relatively small plane‐wave basis set without compromising the computational accuracy. A kinetic energy cutoff of 800 eV and dense Monkhorst–Pack^[^ ^26g^ ^]^ *k*‐point meshes spanning less than 0.015 Å^3^ in the Brillouin zone were chosen.

Due to the discontinuity of exchange‐correlation in the framework of standard DFT, the calculated bandgap was usually smaller than the experimental value, and so a scissor operator^[^ ^27a^ ^]^ was used to shift the conduction bands to match the values. Based on the scissor‐corrected electron band structure, the imaginary part of the dielectric constants can be calculated by the electron transition from the VBs to the CBs. Accordingly, the real part of the dielectric constant, that is, the refractive index, can be determined by a Kramers–Kronig transform of the calculated imaginary part.^[^ ^27b^ ^]^ The refractive indices *n* and the birefringence Δ*n* were thereby obtained. The anisotropic SHG coefficient tensors were calculated.^[^ ^21^, ^27^ ^]^ To gain insight into the contribution of the constituent groups to the SHG coefficient, SHG‐weighted electron density and real‐space atom‐cutting analyses were performed. In SHG‐weighted electron density analysis, the electron density of all the orbitals was summed by a weight positively correlated with its contribution to the SHG coefficient, and thus the electron cloud of orbitals crucial to the SHG response was highlighted in real space.^[^ ^27d^ ^]^ In real‐space atom‐cutting analysis, the contribution of each NLO‐active unit to the SHG coefficient was obtained by excluding the wave functions in a sphere with a radius encompassing all atoms except the focused unit, that is, *χ* ^2^(A) = *χ* ^2^(all atoms excluded except the focused unit).^[^ ^27c^ ^]^

[CCDC 2 286 015 contains the supplementary crystallographic data for this paper. These data can be obtained free of charge from The Cambridge Crystallographic Data Centre via www.ccdc.cam.ac.uk/data_request/cif.].

## Conflict of Interest

The authors declare no conflict of interest.

## Supporting information

Supporting Information

Supporting Information

## Data Availability

The data that support the findings of this study are available in the supplementary material of this article.
