# Supplementary material for: Giant Optical Anisotropy in a Covalent Molybdenum Tellurite via Oxyanion Polymerization
Source: Adv Sci (Weinh). 2024 Jan 30;11(12):2306670. doi: 10.1002/advs.202306670 (PMC10966567; doi:10.1002/advs.202306670)
Supplement: Supplementary file 1 — Supporting Information [file ADVS-11-2306670-s002.pdf]

ADVANCED SCIENCE
Open Access

Supporting Information

for *Adv. Sci.*, DOI 10.1002/advs.202306670

Giant Optical Anisotropy in a Covalent Molybdenum Tellurite via Oxyanion Polymerization

Tianhui Wu, Xingxing Jiang, Kaining Duanmu, Chao Wu, Zheshuai Lin, Zhipeng Huang, Mark G. Humphrey and Chi Zhang**

Supporting Information

Giant Optical Anisotropy in a Covalent Molybdenum Tellurite *via* Oxyanion Polymerization

Tianhui Wu, Xingxing Jiang, Kaining Duanmu, Chao Wu, Zheshuai Lin, Zhipeng Huang, Mark G. Humphrey, and Chi Zhang**

Section	Title	Page
Table S1	Crystallographic data and structure refinement parameters for $Mo(H_2O)Te_2O_7$.	S3
Table S2	Atomic coordinates (× 10^4), equivalent isotropic displacement parameters (Å^2 × 10^3), and the bond valence sum for each atom in the asymmetric unit of $Mo(H_2O)Te_2O_7$. U(eq) is defined as one third of the trace of the orthogonalized U_{ij} tensor.	S4
Table S3	Selected bond distances (Å) and angles (°) for $Mo(H_2O)Te_2O_7$.	S5
Table S4	Hydrogen-bonding interactions for $Mo(H_2O)Te_2O_7$.	S6
Table S5	Calculated linear and nonlinear optical properties for $Mo(H_2O)Te_2O_7$ deduced from real-space atom-cutting analyses[a].	S7
Table S6	Calculated θ (the angle between the direction of the maximum dipole moment component and the direction of the total dipole moment of the [TeO_x] units) for optical d^0-TM tellurites.	S8
Figure S1	Experimental and simulated powder X-ray diffraction patterns of $Mo(H_2O)Te_2O_7$.	S9
Figure S2	Energy dispersive spectroscopy for $Mo(H_2O)Te_2O_7$.	S10
Figure S3	Thermogravimetric analyses of $Mo(H_2O)Te_2O_7$ under a N_2 atmosphere.	S11
Figure S4	UV/Vis transmittance spectrum of $Mo(H_2O)Te_2O_7$ single crystals (inset: crystals for measurement).	S12
Figure S5	Infrared spectrum of $Mo(H_2O)Te_2O_7$.	S13
Figure S6	(a) Asymmetric unit of $Mo(H_2O)Te_2O_7$. (b) [MoO_6] unit. (c) Arrangement of the [MoO_6] units within a unit cell in MTO.	S14
Figure S7	Calculated refractive indices of $Mo(H_2O)Te_2O_7$ crystals with the shortest type-I SHG phase-matching wavelengths marked.	S15
Figure S8	Calculated band structure of $Mo(H_2O)Te_2O_7$.	S16
Figure S9	(a) Oscilloscope traces of the SHG signals for powders of $Mo(H_2O)Te_2O_7$ (105−150 μm). (b) Phase-matching curves of $Mo(H_2O)Te_2O_7$ and KDP with 1064 nm laser radiation. KDP was used as reference for the SHG measurements with 1064 nm laser radiation.	S17
Figure S10	SHG-weighted densities for (a) unoccupied and (b) occupied electronic states in $Mo(H_2O)Te_2O_7$.	S18
References		S19

Table S1. Crystallographic data and structure refinement parameters for $Mo(H_2O)Te_2O_7$.

formula	$Mo(H_2O)Te_2O_7$
formula weight	481.16
temperature (K)	293(2)
crystal system	Tetragonal
space group	$I\bar{4}$ (No. 82)
a (Å)	16.6947(6)
b (Å)	16.6947(6)
c (Å)	5.3133(3)
α (°)	90
β (°)	90
γ (°)	90
V (Å^3)	1480.89(11)
Z	8
ρ_{calc} (g cm^{-3})	4.316
μ (mm^{-1})	9.498
F (000)	1696
θ (deg.)	3.31−27.12
limiting indices	$-21 \leq h \leq 21$,<br>$-21 \leq k \leq 17$,<br>$-6 \leq l \leq 6$
R_{int}	0.0337
no. of reflections (collected/unique)	4566/1639
goodness of fit on F^2	1.121
Absolute structure parameter	0.06(4)
R_1, wR_2 [$I > 2\sigma(I)$][a]	0.0243/0.0619
R_1, wR_2 (all data)	0.0248/0.0623
largest difference peak and hole (e Å^{-3})	1.433 and −1.081

[a] $R_1 = \sum||F_o| - |F_c||/\sum|F_o|$; $wR_2 = [\sum w(F_o^2 - F_c^2)^2/\sum w(F_o^2)^2]^{1/2}$

Table S2. Atomic coordinates (× 10^4), equivalent isotropic displacement parameters (Å^2 × 10^3), and the bond valence sum for each atom in the asymmetric unit of $Mo(H_2O)Te_2O_7$. U_{eq} is defined as one third of the trace of the orthogonalized U_{ij} tensor.

Atom	*x*	*y*	*z*	U_{eq} (Å^2)	BVS
Te(1)	1587(3)	2683(3)	3721(10)	13(2)	3.97
Te(2)	83(1)	3729(1)	711(1)	12(1)	3.93
Mo(1)	2404(1)	906(1)	5059(1)	12(1)	6.39
O(1)	1343(4)	983(5)	7953(14)	32(2)	0.30[a], 2.30[b]
O(2)	2028(4)	−21(4)	4423(14)	29(2)	1.73
O(3)	857(4)	2809(4)	989(13)	18(1)	2.11
O(4)	−701(4)	2991(4)	1982(11)	19(1)	2.25
O(5)	408(4)	4108(4)	3891(12)	16(1)	1.86
O(6)	3232(4)	963(4)	3333(12)	25(2)	1.94
O(7)	2477(3)	2922(3)	1711(12)	15(1)	2.12
O(8)	1650(3)	1553(3)	3048(12)	15(1)	2.00

[a] BVS of O without H atoms. [b] BVS with H atoms and following geometry optimization.

Table S3. Selected bond distances (Å) and angles (°) for $Mo(H_2O)Te_2O_7$.

Te(1)-O(7)	1.873(6)	Te(1)-O(3)	1.907(6)
Te(1)-O(8)	1.922(6)		
Te(2)-O(5)	1.884(6)	Te(2)-O(4)	1.920(6)
Te(2)-O(3)	2.014(6)	Te(2)-O(5)#2	2.183(6)
Mo(1)-O(6)	1.662(6)	Mo(1)-O(2)	1.704(7)
Mo(1)-O(4)#3	1.884(6)	Mo(1)-O(8)	1.972(6)
Mo(1)-O(7)#1	2.153(6)	Mo(1)-O(1)	2.349(8)
O(6)-Mo(1)-O(2)	104.4(3)	O(6)-Mo(1)-O(4)#3	92.2(3)
O(2)-Mo(1)-O(4)#3	101.1(3)	O(6)-Mo(1)-O(8)	101.5(3)
O(2)-Mo(1)-O(8)	98.9(3)	O(4)#3-Mo(1)-O(8)	152.0(3)
O(6)-Mo(1)-O(7)#1	95.5(3)	O(2)-Mo(1)-O(7)#1	160.1(3)
O(4)#3-Mo(1)-O(7)#1	77.1(2)	O(8)-Mo(1)-O(7)#1	77.4(2)
O(6)-Mo(1)-O(1)	170.2(3)	O(2)-Mo(1)-O(1)	84.4(3)
O(4)#3-Mo(1)-O(1)	81.7(3)	O(8)-Mo(1)-O(1)	81.0(3)
O(7)#1-Mo(1)-O(1)	75.7(2)		

[a] Symmetry codes: #1 -x+1/2, -y+1/2, z+1/2; #2 y-1/2, -x+1/2, -z+1/2; #3 y, -x, -z+1.

Table S4. Hydrogen-bonding interactions for $Mo(H_2O)Te_2O_7$.

D-H···A	*d*(D-H)	*d*(H···A)	*d*(D···A)	DHA)
O1-H1A···O2	0.853	2.232	3.084	177.82
O1-H1B···O8	0.855	2.063	2.916	175.52

Table S5. Calculated linear and nonlinear optical properties for $Mo(H_2O)Te_2O_7$ deduced from real-space atom-cutting analyses[a].

		Total	$[Te(1)O_3]$	$[Te(2)O_4]$	$[MoO_6]$	H_2O
$Mo(H_2O)Te_2O_7$	n_o	2.49004	1.78957	1.82369	1.62587	1.23658
	n_e	2.13822	1.57599	1.62501	1.53429	1.21300
	Δn @ 546 nm	0.35182	0.21358	0.19868	0.09158	0.02358
	d_{14}	4.28	2.69	1.98	2.11	0.12
	d_{15}	−2.34	−1.63	−1.29	0.56	0.06

[a] *d* values in pm/V.

Table S6. Calculated θ (the angle between the direction of the maximum dipole moment component and the direction of the total dipole moment of the $[TeO_x]$ units) for optical d^0-TM tellurites.

Compounds	θ (°)
$PbVTeO_5F$[S1]	45.9
$LiNbTeO_5$[S2]	21.0, 31.9
α-$BaTeMo_2O_9$[S3]	29.9, 30.2
β-$BaTeMo_2O_9$[S4]	22.96
α-BaW_2TeO_9[S5]	7.76
$Cs_2TeMo_3O_{12}$[S6]	0
$Cs_2TeW_3O_{12}$[S7]	0
$RbTeMo_2O_8F$[S8]	12.43
Zn_2MoTeO_7[S9]	0
$CdTeMoO_6$[S10]	0
$Cd_3WTe_2O_{10}$[S11]	17.68, 44.78
$Na_2W_2TeO_9$[S12]	11.85, 26.75, 31.19, 36.95
$Na_6W_6Te_4O_{29}$[S12]	25.26, 36.83, 46.63
$Mo(H_2O)Te_2O_7$[this work]	22.7, 23.6

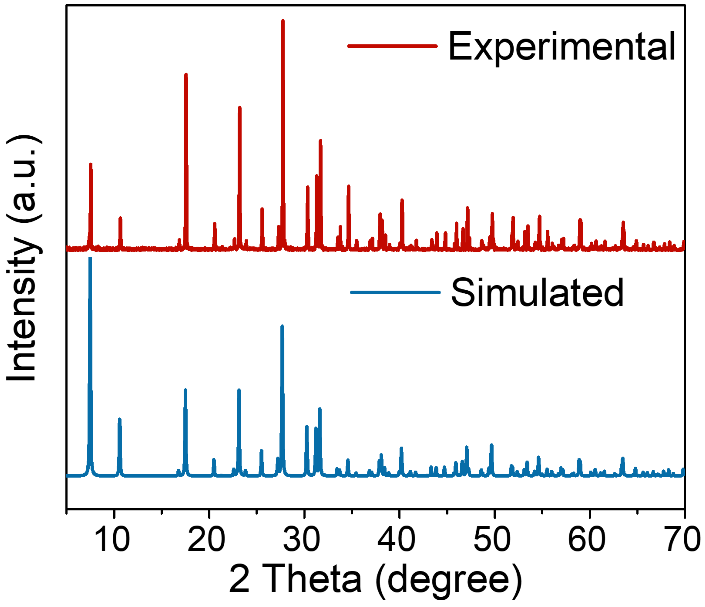

Figure S1. Experimental and simulated powder X-ray diffraction patterns of $Mo(H_2O)Te_2O_7$.

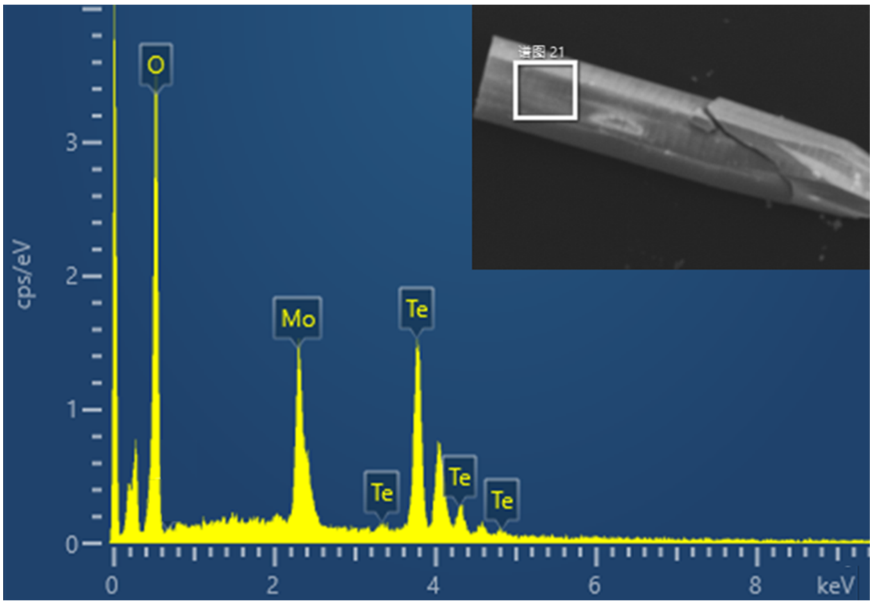

Figure S2. Energy dispersive spectroscopy for $Mo(H_2O)Te_2O_7$.

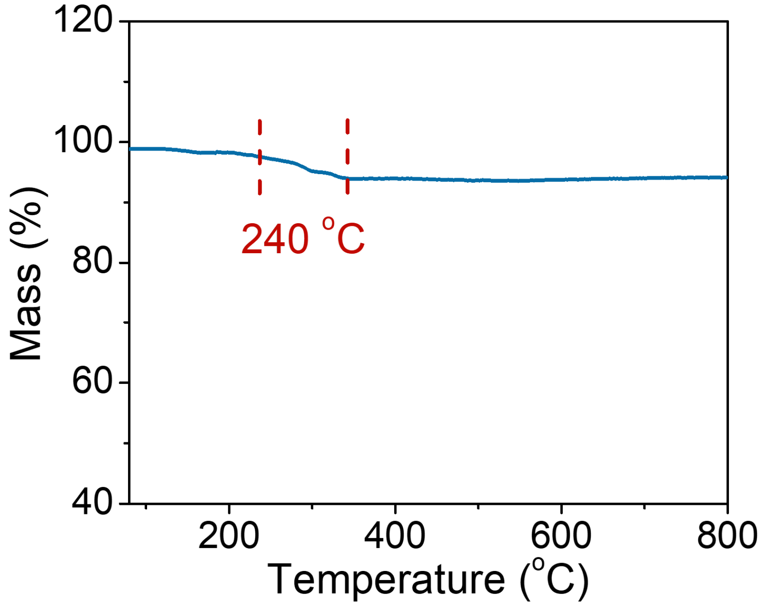

Figure S3. Thermogravimetric analyses of $Mo(H_2O)Te_2O_7$ under a N_2 atmosphere.

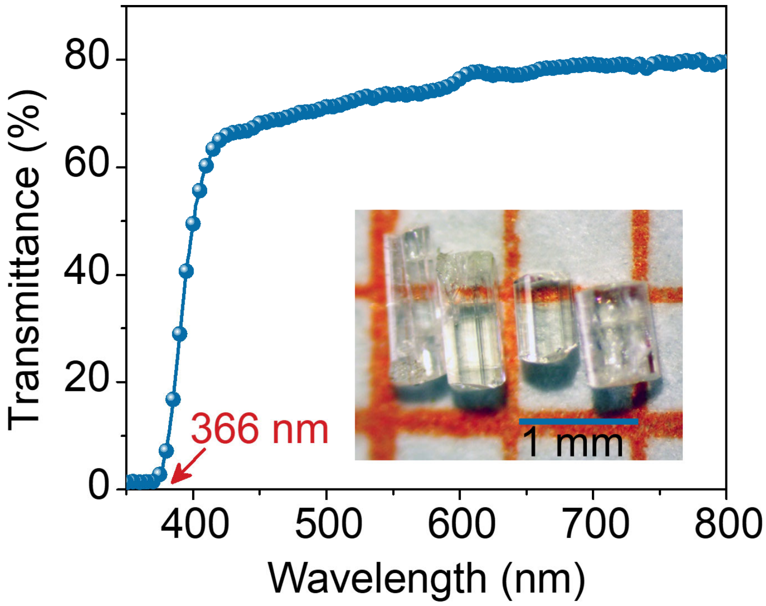

Figure S4. UV/Vis transmittance spectrum of $Mo(H_2O)Te_2O_7$ single crystals (inset: crystals for measurement).

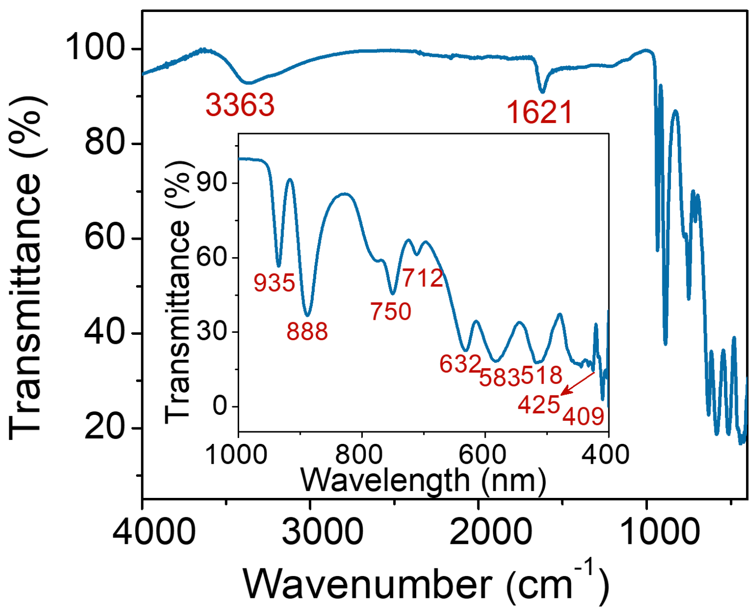

Figure S5. Infrared spectrum of $Mo(H_2O)Te_2O_7$.

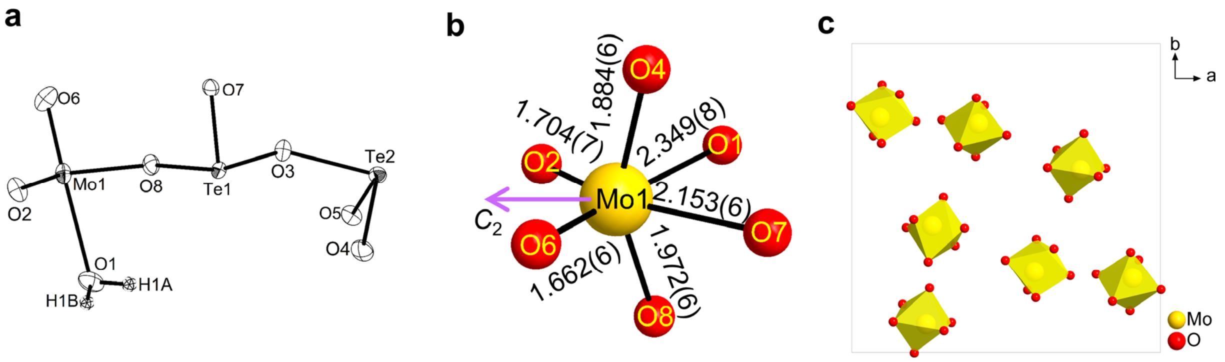

Figure S6. (a) Asymmetric unit of $Mo(H_2O)Te_2O_7$. (b) $[MoO_6]$ unit. (c) Arrangement of the $[MoO_6]^{6-}$ units within a unit cell of MTO.

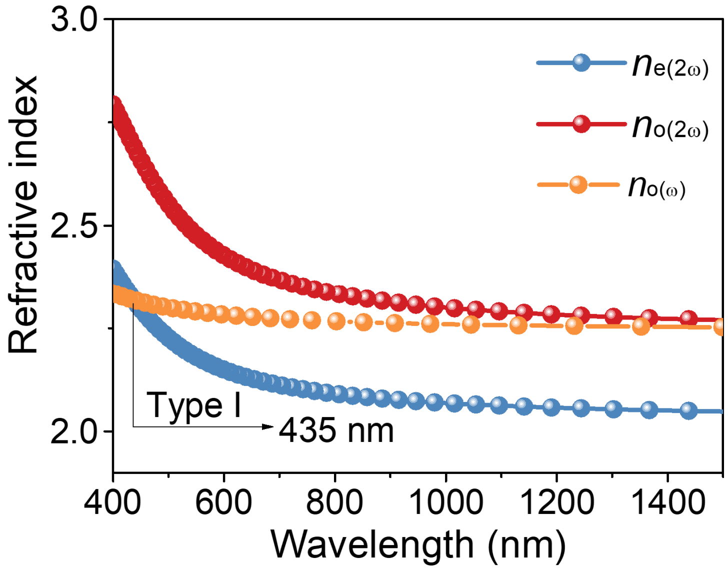

Figure S7. Calculated refractive indices of MTO crystals with the shortest type-I SHG phase-matching wavelengths marked.

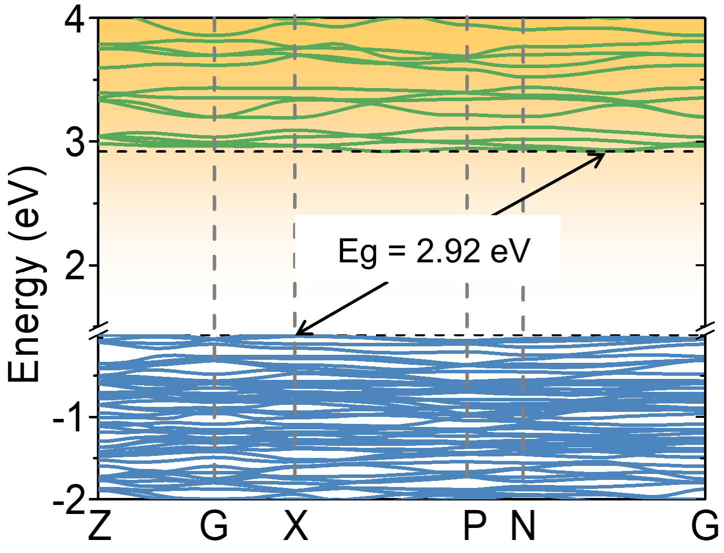

Figure S8. Calculated band structure of $Mo(H_2O)Te_2O_7$.

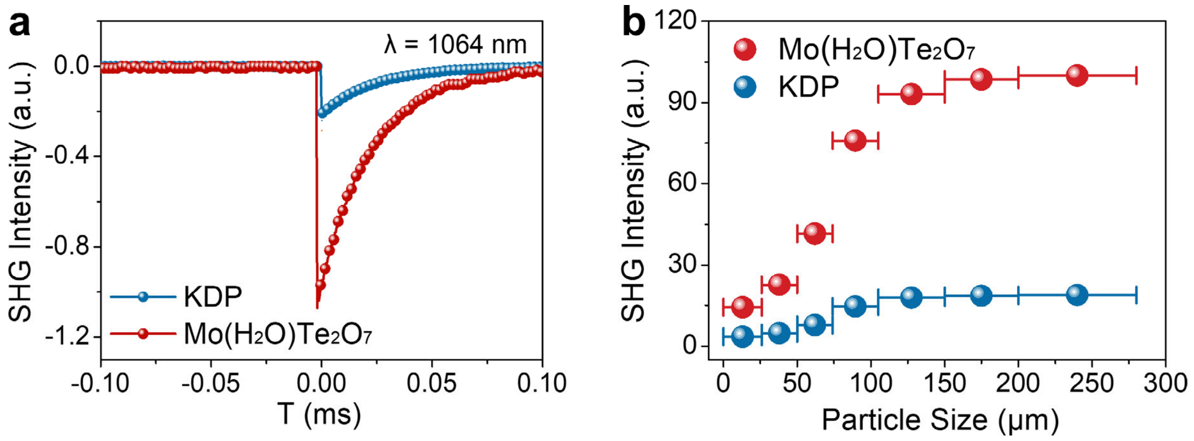

Figure S9. (a) Oscilloscope traces of the SHG signals for powders of Mo(H_2O)Te_2O_7 (105−150 μm). (b) Phase-matching curves of Mo(H_2O)Te_2O_7 and KDP with 1064 nm laser radiation. KDP was used as reference for the SHG measurements with 1064 nm laser radiation.

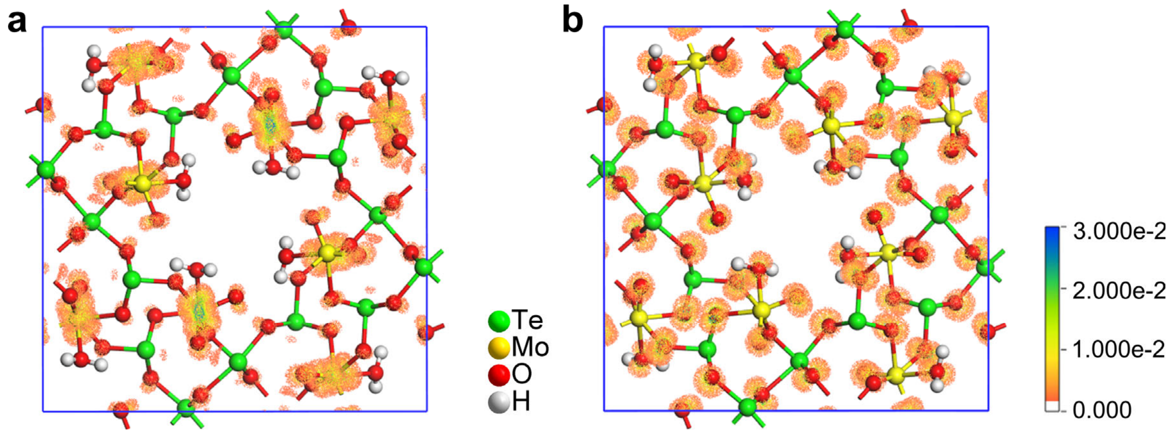

Figure S10. SHG-weighted densities for (a) unoccupied and (b) occupied electronic states in $Mo(H_2O)Te_2O_7$.

References

[S1] Y. Y. Yang, Y. Guo, B. B. Zhang, T. Wang, Y. G. Chen, X. H. Hao, X. X. Yu, X. M. Zhang, *Inorg. Chem.* **2022**, *61*, 1538-1545.

[S2] K. C. Chen, C. S. Lin, G. Peng, Y. Chen, H. Z. Huang, E. Z. Chen, Y. X. Min, T. Yan, M. Luo, N. Ye, *Chem. Mater.* **2022**, *34*, 399-404.

[S3] J. J. Zhang, Z. H. Zhang, Y. X. Sun, C. Q. Zhang, X. T. Tao, *CrystEngComm* **2011**, *13*, 6985-6990.

[S4] W. G. Zhang, X. T. Tao, C. Q. Zhang, Z. L. Gao, Y. Z. Zhang, W. T. Yu, X. F. Cheng, X. S. Liu, M. H. Jiang, *Cryst. Growth Des.* **2008**, *8*, 304-307.

[S5] Z. H. Zhang, X. T. Tao, J. J. Zhang, Y. X. Sun, C. Q. Zhang, B. Li, *CrystEngComm* **2013**, *15*, 10197-10204.

[S6] X. X. Feng, J. J. Zhang, Z. L. Gao, S. J. Zhang, Y. X. Sun, X. T. Tao, *Appl. Phys. Lett.* **2014**, *104*, 081912.

[S7] P. Zhao, Q. Wu, C. L. Li, S. J. Zhang, Y. X. Sun, C. Q. Zhang, S. Q. Xia, Z. L. Gao, X. T. Tao, *Opt. Mater. Express* **2016**, *6*, 451-458.

[S8] Y. L. Hu, C. Wu, X. X. Jiang, Z. J. Wang, Z. P. Huang, Z. S. Lin, X. F. Long, M. G. Humphrey, C. Zhang, *J. Am. Chem. Soc.* **2021**, *143*, 12455-12459.

[S9] W. G. Zhang, P. S. Halasyamani, *J. Solid State Chem.* **2016**, *236*, 32-38.

[S10] C. G. Li, X. X. Tian, Z. L. Gao, Q. Wu, P. Zhao, Y. X. Sun, C. Q. Zhang, S. J. Zhang, D. L. Cui, X. T. Tao, *Cryst. Growth Des.* **2018**, *18*, 3376-3384.

[S11] Q. X. Zhang, Q. Wu, J. F. Zhou, C. Tang, M. J. Xia, *Cryst. Growth Des.* **2022**, *22*, 6678-6685.

[S12] H. W. Yu, W. G. Zhang, P. S. Halasyamani, *Cryst. Growth Des.* **2016**, *16*, 1081-1087.
