# Supplementary material for: Giant Optical Anisotropy in a Covalent Molybdenum Tellurite via Oxyanion Polymerization
Source: Adv Sci (Weinh). 2024 Jan 30;11(12):2306670. doi: 10.1002/advs.202306670 (PMC10966567; doi:10.1002/advs.202306670)

checkCIF/PLATON report

Structure factors have been supplied for datablock(s) 1

THIS REPORT IS FOR GUIDANCE ONLY. IF USED AS PART OF A REVIEW PROCEDURE FOR PUBLICATION, IT SHOULD NOT REPLACE THE EXPERTISE OF AN EXPERIENCED CRYSTALLOGRAPHIC REFEREE.

No syntax errors found. CIF dictionary Interpreting this report

Datablock: 1

```
Bond precision:   Te- O = 0.0064 A         Wavelength=0.71073

Cell:              a=16.6947(6)      b=16.6947(6)      c=5.3133(3)
                   alpha=90          beta=90           gamma=90
Temperature:       293 K

                Calculated                  Reported
Volume          1480.89(14)                 1480.89(11)
Space group     I -4                        I-4
Hall group      I -4                        ?
Moiety formula  H2 Mo O8 Te2                ?
Sum formula     H2 Mo O8 Te2                H2 Mo O8 Te2
Mr              481.16                      481.16
Dx,g cm-3       4.316                       4.316
Z               8                           8
Mu (mm-1)       9.498                       9.498
F000            1696.0                      1696.0
F000'           1674.63
h,k,lmax        21,21,6                     21,21,6
Nref            1648[ 916]                  1639
Tmin,Tmax       0.418,0.514                 0.390,0.746
Tmin'           0.254

Correction method= # Reported T Limits: Tmin=0.390 Tmax=0.746
AbsCorr = MULTI-SCAN

Data completeness= 1.79/0.99       Theta(max)= 27.100

R(reflections)= 0.0243( 1622)                        wR2(reflections)=
                                                     0.0623( 1639)
S = 1.121                    Npar= 101
```

The following ALERTS were generated. Each ALERT has the format
test-name_ALERT_alert-type_alert-level.
Click on the hyperlinks for more details of the test.

Alert level C

```
PLAT199_ALERT_1_C Reported _cell_measurement_temperature ..... (K)        293 Check
PLAT200_ALERT_1_C Reported   _diffrn_ambient_temperature ..... (K)        293 Check
PLAT430_ALERT_2_C Short Inter D...A Contact  O4       ..O6       .       2.89 Ang.
                                                  -y,x,-z  =      4_555 Check
PLAT601_ALERT_2_C Unit Cell Contains Solvent Accessible VOIDS of .         54 Ang**3
PLAT911_ALERT_3_C Missing FCF Refl Between Thmin & STh/L=    0.600          2 Report
PLAT934_ALERT_3_C Number of (Iobs-Icalc)/Sigma(W) > 10 Outliers ..          1 Check
PLAT975_ALERT_2_C Check Calcd Resid. Dens.  0.41Ang From O6      .       1.39 eA-3
PLAT975_ALERT_2_C Check Calcd Resid. Dens.  0.44Ang From O4      .       1.19 eA-3
PLAT975_ALERT_2_C Check Calcd Resid. Dens.  1.01Ang From O7      .       0.49 eA-3
```

Alert level G

```
PLAT002_ALERT_2_G Number of Distance or Angle Restraints on AtSite          4 Note
PLAT004_ALERT_5_G Polymeric Structure Found with Maximum Dimension          1 Info
PLAT005_ALERT_5_G No Embedded Refinement Details Found  in the CIF     Please Do !
PLAT007_ALERT_5_G Number of Unrefined Donor-H Atoms .............          2 Report
PLAT083_ALERT_2_G SHELXL Second Parameter in WGHT  Unusually Large      16.99 Why ?
PLAT093_ALERT_1_G No s.u.'s on H-positions, Refinement Reported as      mixed Check
PLAT152_ALERT_1_G The Supplied and Calc. Volume s.u. Differ by ...          3 Units
PLAT432_ALERT_2_G Short Inter X...Y Contact  Te2      ..O6       .       2.89 Ang.
                                                  -y,x,-z  =      4_555 Check
PLAT794_ALERT_5_G Tentative Bond Valency for Te2       (IV)      .       3.93 Info
PLAT860_ALERT_3_G Number of Least-Squares Restraints ............          3 Note
PLAT899_ALERT_4_G SHELXL-97  is Deprecated and Succeeded by SHELXL     2019/3 Note
PLAT910_ALERT_3_G Missing # of FCF Reflection(s) Below Theta(Min).          2 Note
```

0 **ALERT level A** = Most likely a serious problem - resolve or explain
0 **ALERT level B** = A potentially serious problem, consider carefully
9 **ALERT level C** = Check. Ensure it is not caused by an omission or oversight
12 **ALERT level G** = General information/check it is not something unexpected

4 ALERT type 1 CIF construction/syntax error, inconsistent or missing data
8 ALERT type 2 Indicator that the structure model may be wrong or deficient
4 ALERT type 3 Indicator that the structure quality may be low
1 ALERT type 4 Improvement, methodology, query or suggestion
4 ALERT type 5 Informative message, check

It is advisable to attempt to resolve as many as possible of the alerts in all categories. Often the minor alerts point to easily fixed oversights, errors and omissions in your CIF or refinement strategy, so attention to these fine details can be worthwhile. In order to resolve some of the more serious problems it may be necessary to carry out additional measurements or structure refinements. However, the purpose of your study may justify the reported deviations and the more serious of these should normally be commented upon in the discussion or experimental section of a paper or in the "special_details" fields of the CIF. checkCIF was carefully designed to identify outliers and unusual parameters, but every test has its limitations and alerts that are not important in a particular case may appear. Conversely, the absence of alerts does not guarantee there are no aspects of the results needing attention. It is up to the individual to critically assess their own results and, if necessary, seek expert advice.

Publication of your CIF in IUCr journals

A basic structural check has been run on your CIF. These basic checks will be run on all CIFs submitted for publication in IUCr journals (*Acta Crystallographica*, *Journal of Applied Crystallography*, *Journal of Synchrotron Radiation*); however, if you intend to submit to *Acta Crystallographica Section C* or *E* or *IUCrData*, you should make sure that full publication checks are run on the final version of your CIF prior to submission.

Publication of your CIF in other journals

Please refer to the *Notes for Authors* of the relevant journal for any special instructions relating to CIF submission.

PLATON version of 06/07/2023; check.def file version of 30/06/2023

Datablock 1 - ellipsoid plot

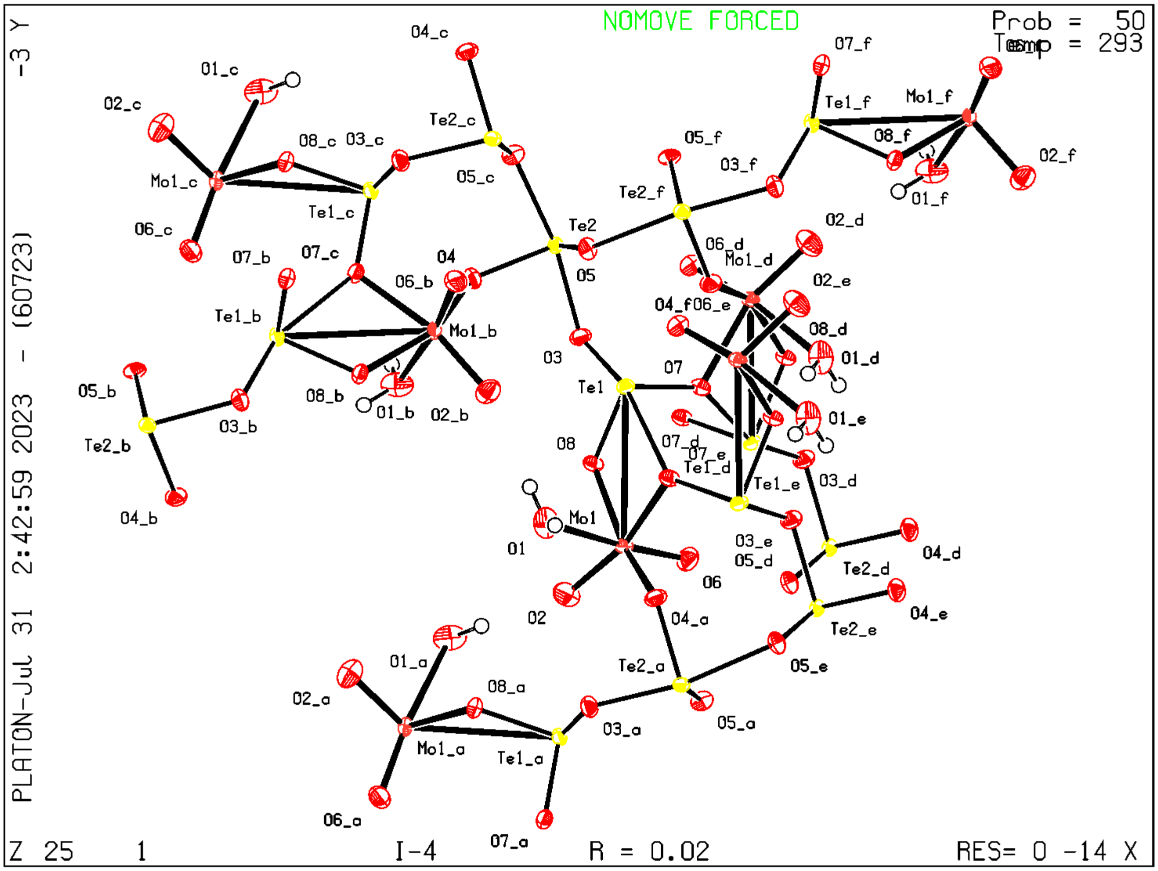

Supplement: Supplementary file 2 — Supporting Information [file ADVS-11-2306670-s001.zip › MTO-CheckCIF.pdf]
